# Effects of High Temperature on Rice Grain Development and Quality Formation Based on Proteomics Comparative Analysis Under Field Warming

**DOI:** 10.3389/fpls.2021.746180

**Published:** 2021-10-21

**Authors:** Wenzhe Liu, Tongyang Yin, Yufei Zhao, Xueqin Wang, Kailu Wang, Yingying Shen, Yanfeng Ding, She Tang

**Affiliations:** ^1^College of Agronomy, Nanjing Agricultural University, Nanjing, China; ^2^Jiangsu Collaborative Innovation Center for Modern Crop Production, Nanjing, China

**Keywords:** rice, global warming, proteomics, data-independent acquisition, grain quality

## Abstract

With the intensification of global warming, rice production is facing new challenges. Field evidence indicates that elevated temperature during rice grain-filling leads to the further deterioration of grain quality. In order to clarify the potential regulatory mechanism of elevated temperature on the formation of rice quality, the DIA mass spectrometry method under the background of field warming was conducted to investigate the regulatory effects of high temperature on grain development and material accumulation pathways. The results showed that a total of 840 differentially expressed proteins were identified during the grain-filling process under elevated temperature. These differentially expressed proteins participated in carbon metabolism, amino acid biosynthesis, signal transduction, protein synthesis, and alternately affected the material accumulation of rice grains. The significant up-regulation of PPROL 14E, PSB28, granule-bound starch synthase I, and the significant down-regulation of 26.7 kDa heat shock protein would lead to the component difference in grain starch and storage proteins, and that could be responsible for the degradation of rice quality under elevated temperature. Results suggested that proteins specifically expressed under elevated temperature could be the key candidates for elucidating the potential regulatory mechanism of warming on rice development and quality formation. In-depth study on the metabolism of storage compounds would be contributed in further proposing high-quality cultivation control measures suitable for climate warming.

## Introduction

With the improvement of people's living standards, high-quality rice is more preferred by the rice production and consumption market. However, with the rapid development of industrialization, human activities are estimated to have caused ~1.0°C of global warming above pre-industrial levels (IPCC, 2018). According to the fifth assessment report (AR5) completed by the IPCC (Intergovernmental Panel on Climate Change), it is estimated that the global temperature is expected to be raised by 1.4–5.8°C in 2100 (IPCC, 2014). The abnormal high temperature would seriously affect the normal growth and development rhythm of rice, and ultimately affect the yield and quality of rice (Jagadish, 2020; Xu et al., 2020). The results of our 10-year field trials showed that rice quality formation generally exhibited negative response characteristics when exposed to elevated temperature. Among them, the increase in temperature have led to the significant increase in chalkiness and the decrease in milling quality of rice, which extremely reduces the purchase expectations and market value of rice (Dou et al., 2017). Therefore, exploring the response mechanism of rice quality under climate change is of great significance for guiding the production of high-quality rice in the future.

As the decisive stage of rice quality formation, grain-filling is the most sensitive period to external temperature. Grain development is accompanied by filling and accumulation of storage substances such as starch, storage protein and lipids, which ultimately determine the relevant indicators of rice quality. As the most abundant components in rice grain, starch had been proved to be sensitive to elevated temperature. Our previous study showed that the accumulation of total starch and amylose in early grain-filling stage was accelerated under the condition of elevated temperature, but the accumulation speed in later stage was significantly decreased, which resulted in the lower content of amylose and total starch in mature grain compared to normal temperature treatment (Tang et al., 2019). In addition, elevated temperature during grain-filling increased the contents of grain storage proteins, with a significantly increased composition of glutelin and decreased prolamin. However, rice with high protein content is more prone to spoilage during storage, and the appearance and eating quality of rice could be further declined (Cao et al., 2017). Furthermore, the activity of protease was enhanced under elevated temperature, and further accelerated the protein transformation into soluble nitrogen compounds, which would significantly increase the total amount of amino acids in rice grains. Overall, elevated temperature mainly accelerated the rate of grain-filling and shortened its active duration, resulting in insufficient accumulation of photosynthetic substances in rice grains (Wahid et al., 2007; Kim et al., 2011). In our previous studies, temperature changes were mainly manifested as abnormal grain development and changes in the accumulation and balance of starch and storage proteins, which synergistically determine the formation of grain quality (Dou et al., 2017; Tang et al., 2018). Although we have obtained the physiological and biochemical evidence of high temperature in regulating grain storage material accumulation through field trials, the regulation mechanism remains to be further elucidated. The synthesis and anabolism of rice starch and proteins is a relatively complex process, including a series of metabolic processes such as synthesis, transport, modification, and accumulation, and it is still difficult to fully grasp the mechanism. Therefore, the main purpose of this study is to further clarify the key regulatory factors involved in grain quality formation under actual paddy field warming scenarios based on the high-throughput proteomics analysis method. An in-depth understanding of the regulation mechanism of climate warming on the synthesis and metabolism of grain storage materials has important practical significance for further establishing high-quality rice cultivation methods under climate warming.

## Results

### Effects of Elevated Temperature on Rice Quality and Accumulation of Grain Storage Materials

An increase of 1.6°-3.1°C in temperature during grain-filling stage induced various changes in the accumulation of storage materials during grain development, and that further affected the rice yield and quality indicators. Results showed that tested rice yield in warming treatment was 21% lower than that of the normal temperature treatment (Table 1). Meanwhile, the elevated temperature increased grain thickness and decreased grain length, and there was no significant change in grain width and aspect ratio of grain shape. Compared with normal temperature, rice chalky rate (36.5%), chalky area (103.3%), and chalkiness (176.4%) were significantly increased under the field warming. Compared with normal temperature, the milled rice rate and head rice rate were significantly reduced by 3.9 and 5.5%. Therefore, based on the field evidence, warming during grain-filling stage led to the overall deterioration in the appearance quality of rice and elevated temperature also had a significant negative effect on rice milling quality.

**Table 1:** Effects of temperature on grain yield and quality traits of rice.

**Treatment**	**Length (mm)**	**Width (mm)**	**Thickness (mm)**	**Length/Width**	**Chalky rate (%)**	**Chalky area (%)**	**Chalkiness (%)**	
CK	4.53a	2.30a	2.07b	1.98a	48.67b	19.43b	9.51b	
ET	4.33b	2.37a	2.13a	1.84a	66.42a	39.50a	26.29a	
**Treatment**	**Total starch (%)**	**Amylopectin (%)**	**Amylose (%)**	**Amylopectin/Amylose**	**Albumin (μg/g)**	**Globulin (μg/g)**	**Prolamin (μg/g)**	**Glutelin (μg/g)**
CK	66.38b	54.95b	11.43a	4.81b	69.7a	65.4a	128.5a	836.3b
ET	70.17a	60.03a	10.14b	5.92a	68.2a	63.8a	113.4b	1092.7a
**Treatment**	**Spikelets per panicle (×10^4^·hm^−2^)**	**Panicles per panicle**	**1,000-grain weight (g)**	**Seed setting rate (%)**	**Yield (** ***t*** **)**	**Brown rice rate (%)**	**Milled rice rate (%)**	**Head rice rate (%)**
CK	471.13a	114.17a	25.03a	95.45a	12.85a	85.90a	76.30a	75.61a
ET	457.11b	104.53b	23.22b	91.47b	10.15b	83.41b	73.31b	71.47b
**Treatment**	**Peak viscosity**	**Hot paste viscosity**	**Breakdown**	**Final viscosity**	**Setback**	**Peak time (min)**	**Pasting temperature (°C)**	
CK	2881b	1573.5b	1498.5b	2466a	−337a	6.1a	73.3b	
ET	3264.5a	1602.5a	1726a	2351b	−924.5b	5.8b	76.7a	

*Values with different letters in the same column are significantly different with p < 0.05; CK, normal temperature; ET, elevated temperature*.

Starch and storage proteins are the main substances that constitute the grain storage material. Under elevated temperature conditions, grain total starches were increased significantly, of which amylopectin was significant increased when responded to high temperature, while amylose was decreased significantly compared with normal temperature treatment. These changes further induced the changes in the proportion of amylopectin/amylose in rice grains. The response of grain storage protein components to elevated temperature showed a significant increase in glutelin and a significant decrease in prolamin. Elevated temperature also induced significant changes in rice cooking quality indicators. Results indicated that the gelatinization characteristics peak viscosity (PKV), hot-paste viscosity (HPV), and gelatinization temperature (GT) were significant increased and the final viscosity was decreased (Table 1). These results suggested that elevated temperature during grain-filling stage have a general negative impact on rice quality parameters.

### Quantitative Expression of Rice Grain Proteins Under Elevated Temperature

**Figure 1:**
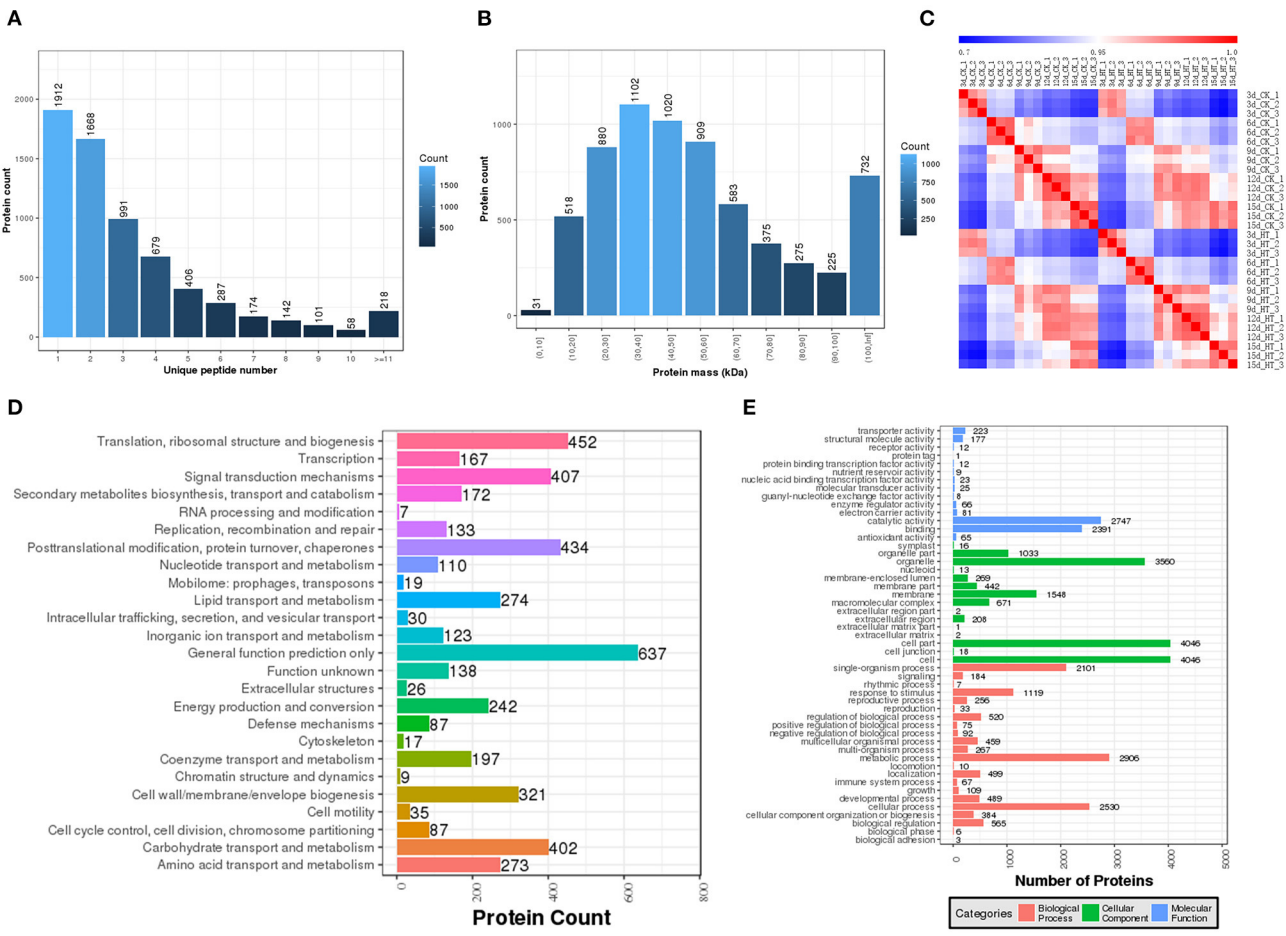
Proteins identified to be responsive to elevated temperature treatment. **(A)** Proteins distributed on the basis of their mass; **(B)** Numbers of unique peptides that were matched to proteins; **(C)** Pearson correlation coefficient; **(D)** GO function classification; **(E)** COG function classification.

In order to further explore the regulation mechanism of elevated temperature on rice quality formation, A quantitative proteomics method was used and results showed that a total of 23,968 unique peptides and 5,872 unique proteins were identified (Supplementary Tables 1–6). The expression and annotation proteins identified in each period were listed and Pearson correlation analysis showed the repeatability of these protein samples was above 97% (Figure 1). Furthermore, proteins were enriched with COG and GO to perform functional analysis, and results showed that the effect of elevated temperature was mainly in regulating the translation, post-translational modification, protein conversion, and signal transduction during the grain-filling stage.

**Figure 2:**
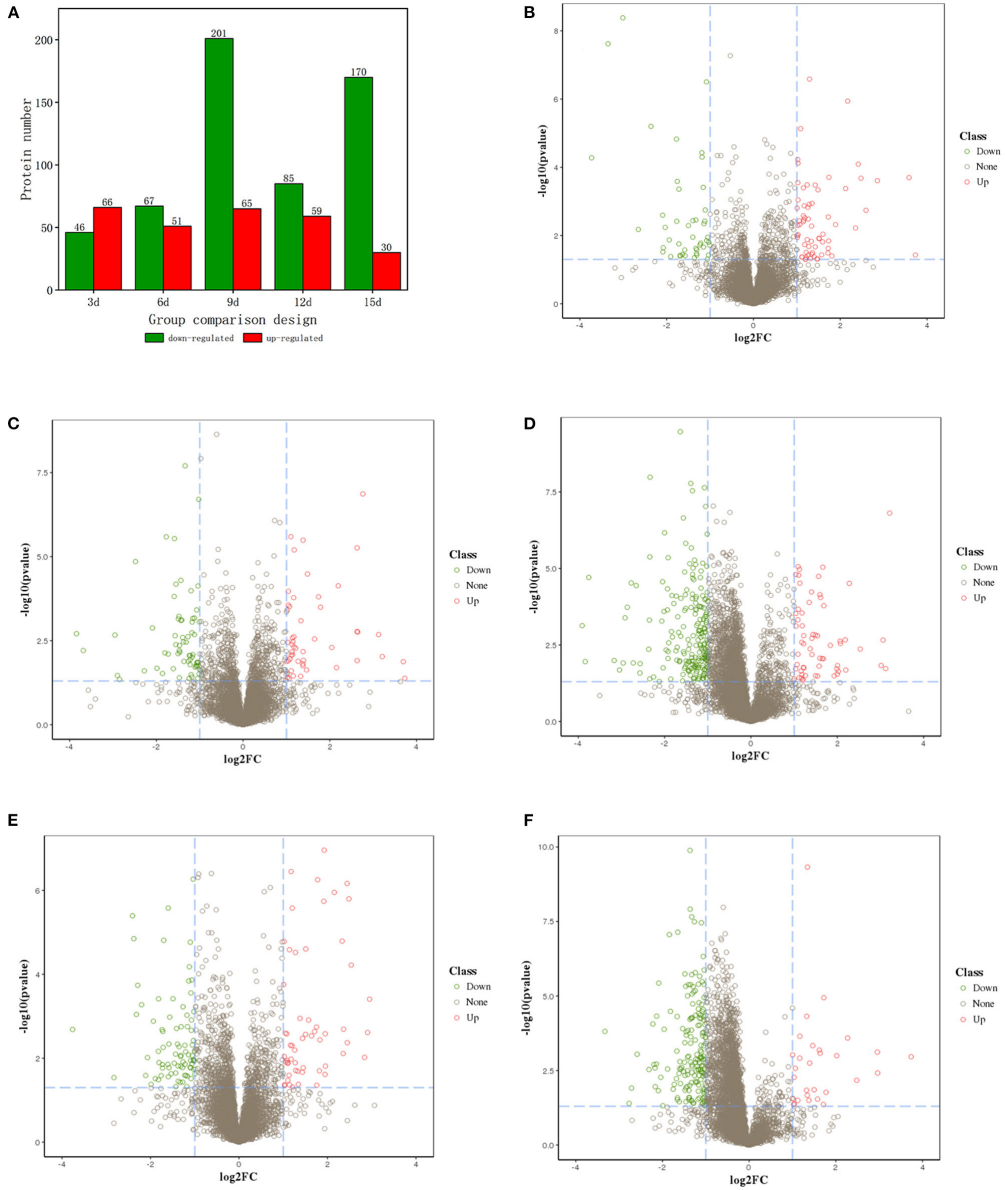
Volcano plots of proteins with differential expression under elevated temperature. **(A)** Number of DEPs in different stages. **(B–F)** Are volcano plots of different expressed proteins on the 3rd, 6th, 9th, 12th, and 15th days after flowering, respectively.

**Figure 3:**
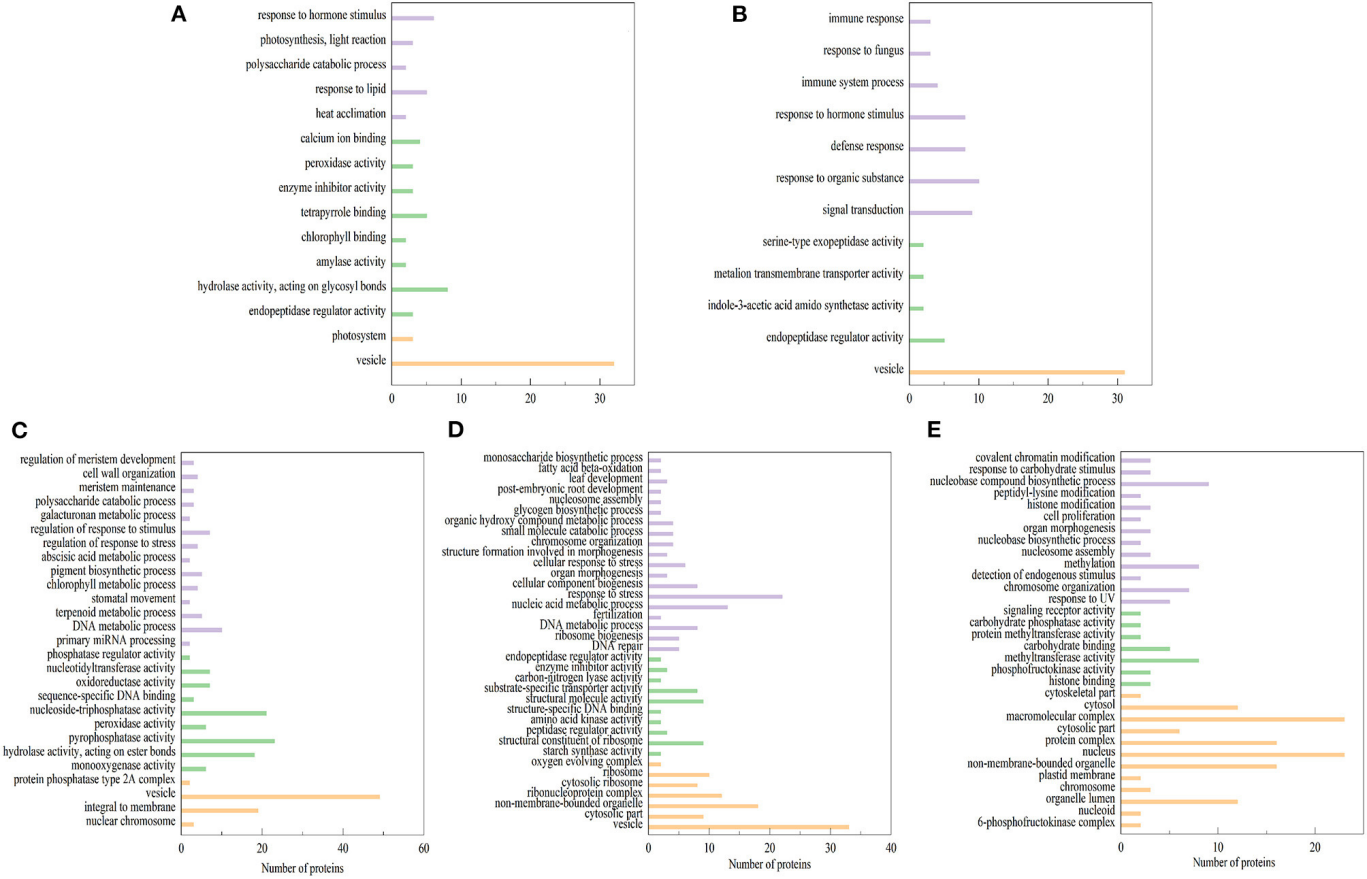
Gene ontology (GO) term enrichment for differentially expressed proteins under elevated temperature. **(A–E)** GO terms (biological process: purple, cellular component: yellow, and molecular function: green) enriched by proteins with differential expression in ET-3d VS CK-3d **(A)**, ET-6d VS CK-6d **(B)**, ET-9d VS CK-9d **(C)**, ET-12d VS CK-12d **(D)**, and ET-15d VS CK-15d **(E)** groups, respectively.

Differentially expressed proteins (DEPs) (Fold change ≥2 and *P* < 0.05) were identified with three biological replicates. Results showed that 112 DEPs were identified in ET-3d (3d after flowering under elevated temperature treatment) and CK-3d (3d after flowering under normal temperature treatment) groups, of which 66 were upregulated and 46 were downregulated. In ET-6d and CK-6d treatments, 118 DEPs were identified, of which 51 were up-regulated and 67 were down-regulated. Comparing to the CK-9d, 65 proteins were up-regulated and 201 proteins were down-regulated in the ET-9d. In ET-12d and CK-12d treatments, 144 DEPs were identified, of which 59 were up-regulated and 85 were down-regulated. In addition, 200 DEPs were found during the 15d after flowering, including 30 up-regulated and 170 down-regulated proteins. The volcano plots and protein annotation of DEPs in ET and CK (3d, 6d, 9d, 12d, and 15d) treatments are shown in Figure 2 and Supplementary Table 2. GO enrichment analysis showed that the prominent GO terms for cellular component enriched by five stages were the vesicle, nucleus, macromolecular complex, non-membrane-bounded organelle, and protein complex. Based on the molecular function, the DEPs were mainly classified into pyrophosphatase activity, hydrolase activity and nucleoside-triphosphatase activity. The top GO molecular function categories were enriched by ET-12d and CK-12d DEPs, including the response to stress, nucleic acid metabolic process and DNA metabolic process (Figure 3).

**Figure 4:**
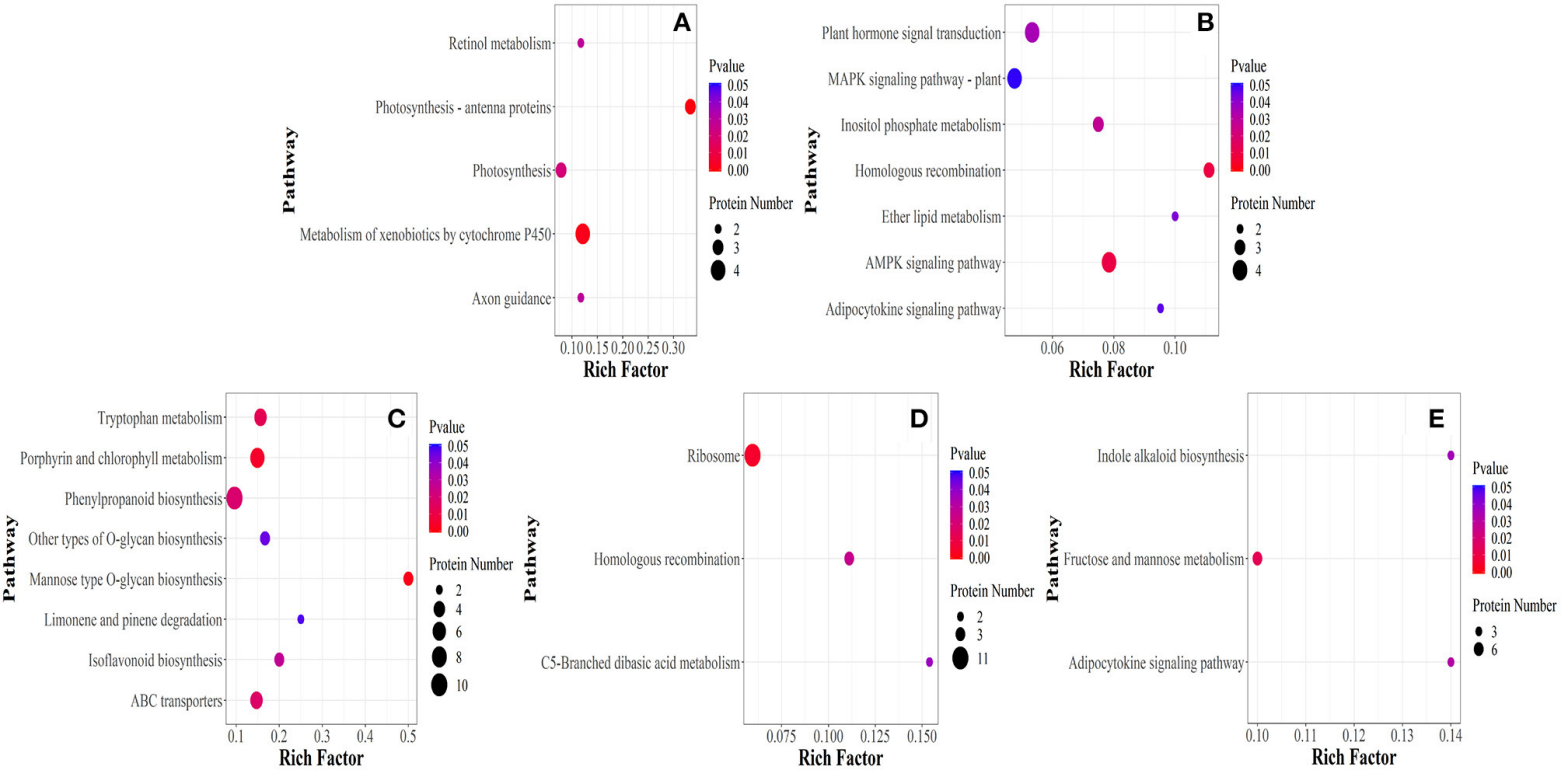
Enrichment of differentially expressed proteins in KEGG pathway under elevated temperature. **(A–E)** Represent the 3rd, 6th, 9th, 12th, and 15th day of samples selected after flowering, respectively.

Differentially expressed proteins were further classified into five stages through KEGG pathway (*P* < 0.05). Metabolic pathways affected under elevated temperature in ET-3d and CK-3d were photosynthesis-antenna proteins, metabolism of xenobiotics by cytochrome P450, photosynthesis, axon guidance, retinol metabolism (Figure 4). In ET-6d and CK-6d, the main pathways were homologous recombination, AMPK signaling pathway, inositol phosphate metabolism, plant hormone signal transduction, ether lipid metabolism, MAPK signaling pathway-plant. Among these, the metabolic pathways enriched in ET-9d and CK-9d mainly include mannose type O-glycan biosynthesis, porphyrin and chlorophyll metabolism, tryptophan metabolism, ABC transporters, phenylpropanoid biosynthesis, isoflavonoid biosynthesis, other types of O-glycan biosynthesis, limonene, and pinene degradation. DEPs identified through pathway enrichment analysis of ET-12d and CK-12d were mainly enriched in ribosome, homologous recombination, C5-Branched dibasic acid metabolism. Furthermore, fructose and mannose metabolism, adipocytokine signaling and indole alkaloid biosynthesis metabolic pathways were found to be more sensitive to elevated temperature at the 15d after flowering, and that indicated the temperature had significantly different regulating effects on different stages of grain development.

### Identification of Differentially Expressed Proteins Related to Rice Development and Quality Formation

**Figure 5:**
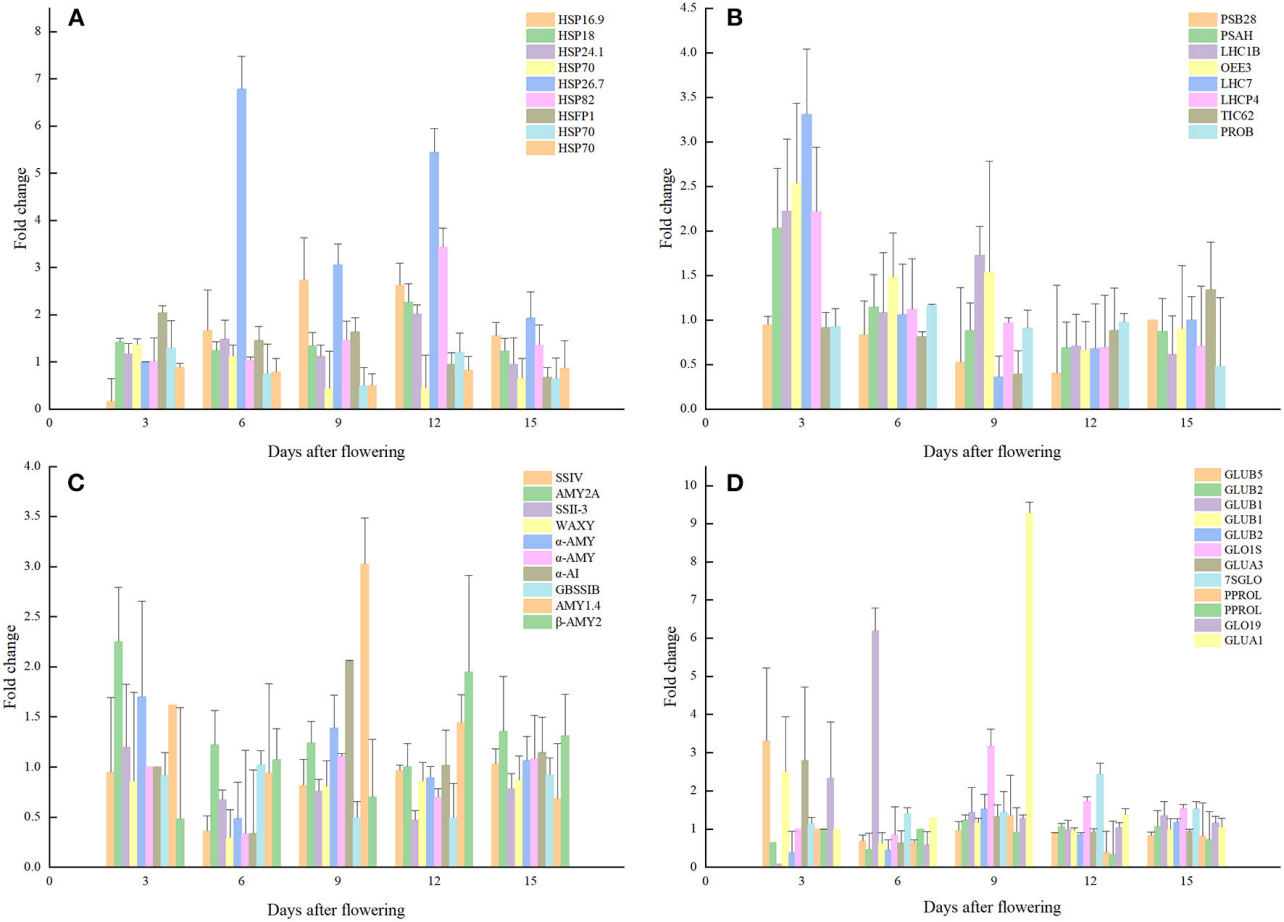
Expression analysis of proteins related to quality formation pathways. **(A)** Storage proteins; **(B)** Molecular chaperone; **(C)** Photosynthesis; **(D)** Starch synthesis.

A total of 748 unique proteins were identified based on their specific expressions when exposed to elevated temperature. After removing 302 proteins with lower scores and unknown functional classification, 39 proteins related to rice development and quality formation were distinguished (Table 2, Figure 5). In this study, the expression of 16.9 kDa class I heat shock protein in rice grains was significantly reduced at the 3d after heating, with a down-regulation of 84% compared with CK treatment. However, the expression of heat shock factor binding protein 1 involved in heat shock protein synthesis was significantly increased. Furthermore, at the early stage of grain filling, the expression of HSP70 decreased significantly, and the expression on the 3rd, 6th, and 9th day after flowering was only 0.47, 0.5, and 0.43 folds that of normal temperature treatment, respectively. Results showed the most significant change was the 26.7 kDa heat shock protein of the sHSPs family, and its expression was increased significantly at 6, 9, and 12 days after flowering, with 6.78, 3.06, and 5.44 folds compared to the CK treatment. Expressions of 18.0 kda class II heat shock protein, 24.1 kda heat shock protein and heat shock protein 82 were up-regulated at the 12d after flowering (2.26, 2.01, 3.63 folds, respectively) when subjected to elevated temperatures.

**Table 2:** Differentially expressed proteins under elevated temperature.

	**Protein_ID**	**PG_Cscore**	**Description**	**Days after flowering**	**Fold change**
Molecular chaperone	1002227700	5.589271	16.9 kDa class I heat shock protein 3	3	0.16
	1002274378	5.026661	Heat shock factor-binding protein 1	3	2.04
	1002249168	5.638757	26.7 kDa heat shock protein, chloroplastic	6; 9; 12	6.78; 3.06; 5.44
	1002279871	5.834035	Heat shock 70 kDa protein 16 isoform X1	9	0.47
	1002296675	5.888584	Heat shock 70 kDa protein, mitochondrial	9	0.5
	1002244395	5.738268	Heat shock 70 kDa protein 17	9; 12	0.43; 0.44
	1002231095	5.419388	18.0 kDa class II heat shock protein	12	2.26
	1002244087	5.593509	24.1 kDa heat shock protein, mitochondrial	12	2.01
	1002259909	5.077947	Heat shock protein 82	12	3.43
Storage protein	1002256234	5.534615	Glutelin type-A 3	3	2.79
	1002242479	5.804829	Glutelin type-B 1	3	2.49
	1002269601	5.323533	19 kDa globulin	3	2.32
	1002238885	5.933567	Glutelin type-B 5-like	3	3.3
	1002239810	5.454091	Glutelin type-B 1-like	3; 6	0.08; 6.19
	1002245900	5.419344	Glutelin type-B 2-like	3; 6	0.38; 0.44
	1002239619	5.638689	Glutelin type-B 2-like	6	0.46
	1002248312	5.772556	Globulin-1 S allele	9	3.17
	1002288955	5.518718	Glutelin type-A 1-like	9	9.27
	1002268046	5.309461	Prolamin PPROL 14E	12	0.38
	1002268263	5.446809	Prolamin PPROL 14E-like	12	0.33
	1002266396	4.918256	Basic 7S globulin	12	2.42
Starch synthesis	1002273855	4.53732	Alpha-amylase isozyme 2A	3	2.25
	1002296409	5.016673	Beta-amylase 2, chloroplastic isoform X1	3	0.48
	1002280365	5.764317	Granule-bound starch synthase 1	6	0.29
	1002230293	4.951311	Probable starch synthase 4	6	0.36
	1002282772	5.383006	Alpha-amylase/trypsin inhibitor	6	0.33
	1002282035	5.501471	Alpha-amylase/trypsin inhibitor	6	0.48
	1002284731	5.634974	Alpha-amylase inhibitor 5	6; 9	0.33; 2.05
	1002292698	5.531977	Alpha-amylase isozyme 3E	9	3.02
	1002285817	5.765962	Granule-bound starch synthase 1b	9; 12	0.5; 0.49
	1002279853	5.673613	Soluble starch synthase 2-3	12	0.47
Photosynthesis	1002280024	5.588999	Chlorophyll a-b binding protein 1B-21	3	2.22
	1002272608	5.337404	Photosystem I reaction center subunit VI	3	2.03
	1002284550	5.787986	Oxygen-evolving enhancer protein 3	3	2.53
	1002290793	4.661148	Chlorophyll a-b binding protein P4	3	2.21
	1002286185	4.714122	Chlorophyll a-b binding protein 7	3; 9	3.3; 0.36
	1002298841	5.009246	Protein TIC 62	9	0.39
	1002224946	4.238946	Photosystem II reaction center PSB28 protein	12	0.4
	1002303125	5.539293	Protochlorophyllide reductase B	15	0.48

Proteins involved in the accumulation of storage protein and starch in rice grains were also significantly affected by the elevated temperature. For example, the glutelin type-A 3, glutelin type-B 1, glutelin type-B 5-like expression levels of rice grains were significantly up-regulated by 2.79, 2.49, and 3.3 folds, respectively. At the same time, the expression levels of glutelin type-B 1-like and glutelin type-B 2-like proteins were decreased significantly. Furthermore, the expression levels of globulin 19 kDa globulin, globulin 1S allele and basic 7S globulin were increased significantly, and that was consistent with the increased content of globulin measured in mature grains. In this study, the significant decrease in prolamin under warming conditions was mainly at the 12d after flowering and expression analysis of prolamin PPROL 14E and prolamin PPROL 14E-like proteins showed that they were both decreased significantly (0.33, 0.38 folds) at this stage when comparted to the normal temperature treatment. As the main component of rice grains, the accumulation of starch has been proved to be sensitive to elevated temperature. Results indicated that enzyme related to starch synthesis during rice starch synthesis were also affected by temperature. In this study, the core starch synthesis-related enzyme GBSS (granule-bound starch synthase) was down-regulated at the 6d after flowering under elevated temperature. While proteins related to amylopectin synthesis obtained no significant changes when compared to the CK. The expression levels of GBSS at 6d, 9d, and 12d after flowering were significantly lower than that of the control and the expression levels of soluble starch synthase responsible for the synthesis of amylopectin SS-IVand SSSII-III were also decreased under the elevated temperature.

Photosynthesis is the process by which light energy is converted into chemical energy and stored, and thus it is essential for the accumulation of rice grain assimilation. Results showed that elevated temperature during the grain filling period had a significant impact on the rice photosynthetic system of rice, which could further affect the formation of the final quality. In this study, the expression levels of the chlorophyll a-b binding protein 1B-21, chlorophyll a-b binding protein P4, and chlorophyll a-b binding protein 7 in chlorophyll ab binding protein were significantly up-regulated at the beginning of grain-filling (2.22, 2.03, 3.3 folds), when compared to the CK. However, the expression of PSB28, which is responsible for water splitting, had a downward trend through the grain-filling stage, and reaching a significant level at 12 days after flowering. Furthermore, TIC 62 (translocon at the inner envelope membrane of chloroplasts) was significantly down-regulated at 9d after flowering, and that may negatively affect the dynamic balance of the proteome in rice grains.

## Discussion

### Assessment of Field Warming and the Impact on Rice Quality Formation

In order to realistically simulate the characteristics of global warming, the free-air temperature enhancement (FATE) facility was installed in the actual paddy field to perform the warming scenarios during the rice grain-filling process. The FATE device was suspended above the field and 12 sets of ceramic infrared heaters were used to perform uniform heating in an area of 7.1 m^2^. The daytime canopy temperature of rice could be increased by FATE with 2.4°C when fully activated on the basis of normal temperature, and the nighttime temperature of canopy could be increased by 5.4°C. This warming range was within the prediction of possible temperature increased by 1.4–5.8°C at the end of the twenty first century by IPCC (2014). Furthermore, field warming effects showed that the increase in night temperature was significantly higher than that during the daytime and that is also consistent with the asymmetric trend of climate warming (Pachauri et al., 2014). Compared with closed or semi-closed warming scenario, the warming method and effect adopted in this study could more authentically simulate climate warming characteristics and that provided a more reliable platform for us to conduct related experiments in the actual field.

Grain filling is the key period for grain quality formation, and it is also the period most sensitive to external temperature. Our field evidence indicated that the increase in temperature generally had the relatively negative impact on rice quality, including the significantly increased chalky rate, chalky area and chalkiness. Meanwhile, the milled rice rate and head rice rate were decreased significantly, and that exceedingly reduced the grain milling quality and the market acceptance of rice. To our knowledge, the changes of external temperature inevitably affected the morphological composition and structure of the grain storage material, and that further induced the changes of related quality traits (Dou et al., 2017, 2018; Tang et al., 2018, 2019). Among these attributes, eating and cooking quality (ECQ) is one of the most important indicators, especially from the consumer's perspective. The eating quality refers to the sensory perception of consumers on rice, and that is related to the gloss, flavor, and viscosity of rice. Although the physical and chemical properties of starch in rice endosperm can be used as an indirect indicator of ECQ, it is still difficult to assess ECQ through these traits. At the same time, the increase of glutelin content in rice grain is particularly obvious under the condition of elevated temperature, and that could further induce the overall balance change of grain storage materials and negatively affect the taste and appearance quality of the rice.

### Overview of DIA Quantitative Proteomics Analysis

In recent years, proteomics-based mass spectrometry has made significant progress from sample preparation to liquid chromatography and instrument detection, making it possible to identify more specific expressed proteins in cells or tissues with excellent accuracy and repeatability (Tsou et al., 2015). Data independent acquisition (DIA) is widely used in proteomics analysis due to its higher protein coverage rate and reliable data acquisition ability (Searle et al., 2015; Renaud and Sumarah, 2016). Compared with iTRAQ, the advantage of DIA technology can effectively measure protein molecules with extremely low-abundance protein molecules in complex samples, which greatly improves the reliability and accuracy of quantitative analysis a. In this study, MaxQuant was used to perform the database search and identification process, and obtained all detectable non-redundant high-quality MS/MS spectral information as DIA spectral library, which contains the fragment ion intensity and retention time describing the peak characteristics of the peptides. We identified 23,968 unique peptides and 5,872 unique proteins, including 840 differential expressed proteins under warming environment. These identified specifically expressed proteins have significant differences in temporal and spatial expression characteristics, which provided the obstacles for us to further identify and screen key regulatory factors. Therefore, this research mainly focused on the essential relationship between grain filling and quality formation, and we further screened the key proteins which were specifically affected by warming during the quality formation process from the perspectives of plant photosynthesis, grain starch, and storage protein accumulations. The results further indicated that induction of the key proteins could lead to the changes in grain storage materials and that could be one of the main reasons for the deterioration of quality under elevated temperature.

### Key Regulatory Factors Contributed to Rice Quality Formation Under Elevated Temperature

Photosynthesis is the process by which light energy is converted into chemical energy and stored, and it is also the source of accumulation of rice grain assimilation. Chlorophyll content and metabolic enzyme activity are closely related to the strength of photosynthesis. In our case, chlorophyll a-b binding protein 1B-21, chlorophyll a-b binding protein P4, and chlorophyll a-b binding protein 7 were significantly up-regulated, which induced the acceleration of the synthesis and binding of chlorophyll (Ballottari et al., 2012). Meanwhile, the expression levels of photosystem I reaction center subunit VI and oxygen-evolving enhancer protein 3 involved in the photosystem I were also increased significantly under warming conditions, and that may explain the accelerated grain filling rate and the significant increase in the accumulation rate of grain materials during the early grain-filling stage under elevated temperature. However, the expression of PSB28, which is responsible for water splitting, had a downward trend throughout the period, obtaining a significant low level at 12d after heading under elevated temperature (0.4 folds). That may inhibit the electron transfer and weaken signal transmission, thereby weakening photochemical reactions and resulting in decreased cell chlorophyll and photosynthesis (Wada et al., 2019). Previous studies have shown that the optical system II (PS II) is the most temperature-sensitive element in the electron transmission chain (Zhou et al., 2013). It would be interesting to further investigate whether PSB28 could be the most critical component affected by elevated temperature during the photosynthesis process.

Photosynthesis is the source of grain assimilate accumulation, and the influence of elevated on photosynthesis will directly lead to changes in the structure and composition of storage materials such as starch in grains. To our knowledge, the contents and ratio of starch and storage protein in rice grains are the decisive factors that determine the final rice quality. Rice starch synthesis is regulated by various proteins and enzymes, including SSS, SBE, DBE, and GBSS. Wx protein encoded by the Waxy gene GBSS-I can tightly bind to the starch granules and promote the synthesis of amylose. Elevated temperatures may induce the down-regulation of gene expression that regulates GBSS synthesis, resulting in decreased amylose content and increased amylopectin content, and the changes of the physical and chemical properties of rice starches have been verified in previous studies (Dian et al., 2005; Fujita et al., 2006; Tang et al., 2019). However, the mechanism of GBSS on the extension of normal starch granules is still unclear. Our results indicated that the GBSS enzyme was down-regulated at 6d after flowering under elevated temperature. However, enzymes related to amylopectin synthesis did not change significantly. From 6d to 12d after flowering, the expression level of granule-bound starch synthase was significantly lower than that of the control. Under warming conditions, the amylose content of mature rice grains was significantly lower compared to the CK, while the amylopectin content was significantly increased. Expression levels of the soluble starch synthase SSIV and SSSII-III, responsible for the synthesis of amylopectin, were also decreased under high temperatures. This change may reduce the activities of granular starch synthase and soluble starch synthase, and lead to change in the ratio of amylose and amylopectin, which eventually affected the physical and chemical properties of starches in rice grain (Hakata et al., 2012; Ahmed et al., 2015; Tang et al., 2019).

Storage proteins are the second largest storage substance in rice grains, accounting for about 8% of the grain dry weight. Rice storage proteins are composed of albumin, globulin, glutelin, and prolamin. Prolamin is directly deposited in the endoplasmic reticulum cavity in the form of intracellular protein particles, and finally buds from the endoplasmic reticulum in the form of spherical protein bodies (PBs). While glutelin is efficiently converted into mature form by vacuolar processing enzymes and forms irregular protein bodies II (PBII) together with α-globulin (Krishnan et al., 1992; Kumamaru et al., 2010). The results of this study showed that warming had significant up or down regulation effects on the expression of storage protein family-related regulatory factors at different stages of grain development. For example, the expression of glutelin type-A and type-B proteins were either significantly up-regulated or down-regulated at 3d and 6d after flowering, and there is no obvious rule for the regulation mode of these regulatory factors under warming conditions. Based on our understanding, the presence of many unknown genes involved in the glutelin synthesis pathway increases the difficulty of understanding expression patterns under warming conditions. Therefore, this study has not been able to essentially identify the regulation mechanism of the changes in the protein content of the final grain storage.

Ribosomes are the primary sites for protein synthesis, and different species of ribosomal proteins play an essential role in translation, ribosome structure, and biogenesis in protein anabolism (Moin et al., 2016a). In this study, the ribosomal protein species (25S, 30S, 40S, 50S, and 60S) exhibited significant decreases during the middle stage of grain-filling, which may cause the reduction in the protein biosynthesis and maintain the balance between synthesis and degradation of proteins (Moin et al., 2016b). The reduction in protein content related to translation, such as RNA recognition motif (RRM) domains, eukaryotic initiation factors (eIFs) and elongation factors (EFs), indicated the adverse effects of elevated temperature on rice protein synthesis. Furthermore, a series of molecular chaperone heat shock proteins (Hsps) were identified to be significantly up-regulated when exposed to elevated temperature. Heat shock proteins are a class of highly conserved peptides in structure and could be activated and produced in large quantities when plants are subjected to abiotic stress (Timperio et al., 2008). In this study, the two most sensitive heat shock proteins are HSP70 and 26.7 kDa heat shock protein from the sHsps (small heat shock proteins) family. Wang et al. (2014) found that overexpression of Hsp70 encoded gene could positively improve the tolerance of plants when subjected to heat stress. In our results, the expression level of HSP70 was decreased sharply at 9d, which in turn led to its inability to participate in the import and translocation of precursor proteins, and that further induced the disorders of rice protein synthesis in rice grains. Li et al. (2019) found that overexpression of Hsf5 could significantly increase the basic heat tolerance of plants in Arabidopsis. The function of sHSP is similar to other ATP-dependent members such as Hsp70, thereby assisting the correct folding and configuration of the protein for further processing (Tabassum et al., 2020). Grain storage protein precursors such as glutelin precursors are synthesized in the endoplasmic reticulum, and then folded and modified with the help of a series of molecular chaperones to form trimers. These trimers are then transported out of the endoplasmic reticulum through vesicles, and eventually transported to protein storage vacuoles to form protein bodies (PB II) (Ren et al., 2020). During this process, the molecular chaperone heat shock protein family such as Hsp70/BiP located at the endoplasmic reticulum can promote the correct folding of glutelin and keeps the protein stable during the folding and assembly process. In this study, HSP70 and HSPS were significantly expressed under elevated temperature and that could promote the correct protein folding during rice grain filling and further contribute to the accumulation of storage proteins in rice grain.

## Materials and Methods

### Experimental Site Description

The experiment was conducted at the Danyang Experimental Base of Nanjing Agricultural University (31°56′39″N, 118°59′13″E, 80 m). The experiment site belongs to the main high-yield rice cultivation area in the lower reaches of the Yangtze River in China. The climate belongs to the subtropical monsoon climate and the soil condition is loam with a pH value of 6.04. The total soil nitrogen was 1.4 g·kg^−1^, the available nitrogen was 7.8 mg·kg^−1^, the available phosphorus was 20.1 mg·kg^−1^ and the available potassium was 91.7 mg·kg^−1^. The temperature and precipitation in the experiment are shown in the Supplementary Figure 1.

### Plant Materials and Temperature Treatment

**Figure 6:**
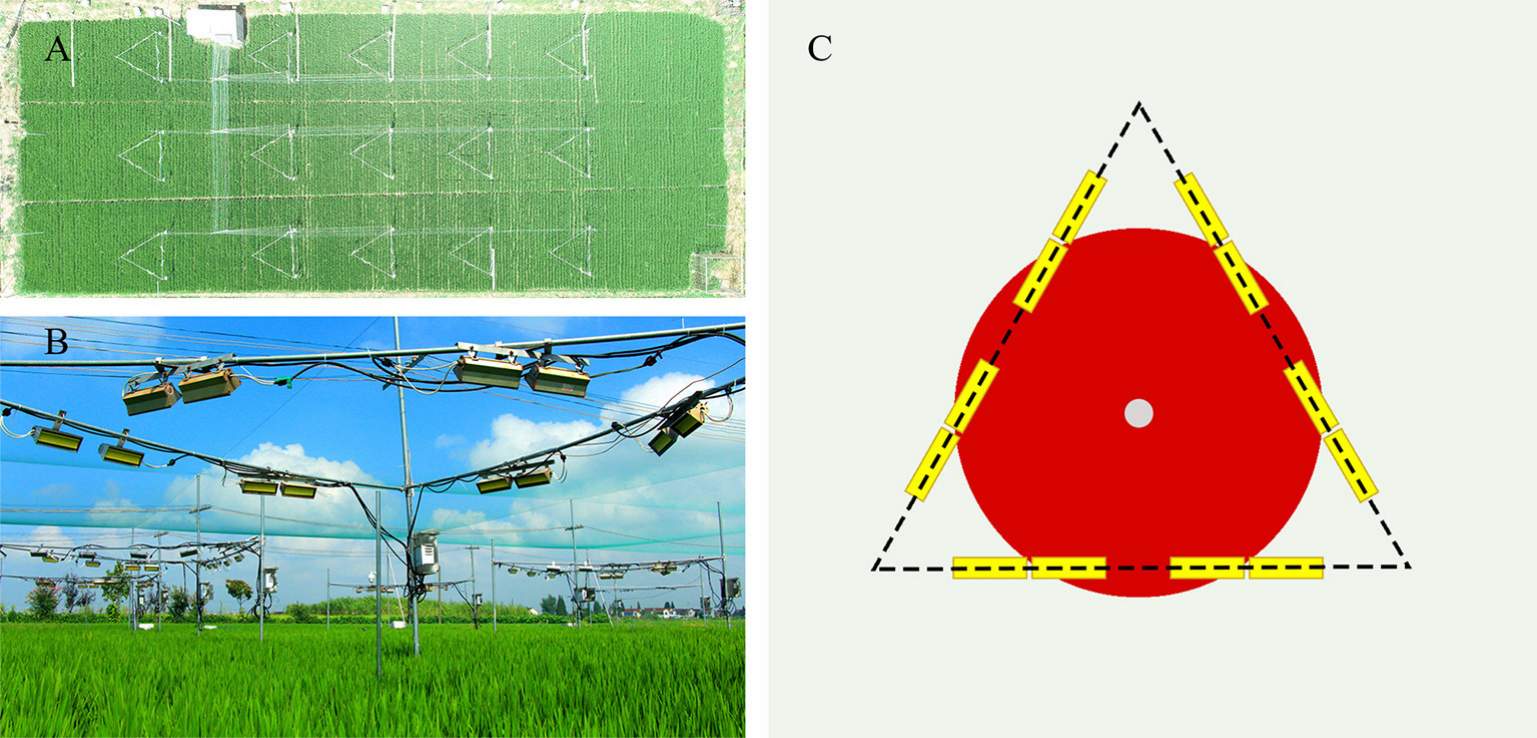
Actual field warming scene based on Free-air temperature enhancement (FATE) system. **(A)** Aerial image of test field block **(B)** Real scene of warming community **(C)** System configuration diagram (The yellow rectangle is an infrared heating device; The white circle is HOBO; The red area is the warming range).

Wuyujing 3 (W3), a high-quality variety that is widely planted locally, was selected as the rice material for this study. Temperature treatments (normal temperature and elevated temperature) were conducted with 3 replicates for each treatment. An interval of 80 cm, and a protection line were set between the treatment blocks to ensure the independence of the experiment. Field cultivation management was conducted according to the local high-yield cultivation measures. The free air warming system (FATE) was used to increase the rice canopy temperature from the rice flowering stage. Twelve ceramic infrared radiator heaters (FTE-1000-240-0-L10-Y; 1000W, 240V) were installed 1.2 m above the rice canopy in each block (length 245 mm × width 60 mm). The heaters were in the horizontal direction and the vertical angles were 45° and 30° to ensure continuous and stable heating. The effective area of infrared radiation was 1.5 × 1.5 × 3.1416 = 7.1 m^2^ (Figure 6). Two sensors were installed at the height of the canopy (HOBO U23-001) to record the temperature and humidity of rice canopy. The field meteorological data during the test was collected from a weather station (WatchDog 550) located at ~100 m away from the test site (Rehmani et al., 2011, 2014; Dou et al., 2018; Tang et al., 2019). According to the meteorological data, rice canopy temperature was increased by 1.568° and 3.089°C during the day and night, respectively (Supplementary Figure 1). The temperature increase range and trend were in line with the characteristics of global warming.

### Sampling

Rice superior spikelets (located at the upper 1/3 of the rice panicle) flowering on the same day were tagged and were collected at 9:00 am on the 3rd, 6th, 9th, 12th, and 15th day after flowering. Thirty marked and representative spikelets were randomly selected and every 10 spikelets were divided into a replicate. Spikelets located at the upper third of the panicle were cut and quickly stored in liquid nitrogen. Glumes of the grains were carefully peeled off using tweezers, and for each replicate about 0.5 g of the grains were used for proteome determination. All samples collected from the ET treatment were within the effective warming area (Figure 6). At the mature stage, 10 holes were randomly selected from each treatment and the number of panicles per hole, spikelets per panicle, 1,000-grain weight, seed setting rate were investigated to calculate the final yield. Starch and protein content were determined using the spikelets air dried under natural conditions.

### Measurement of Starch and Storage Protein Composition

Refined rice samples were milled into flour in the liquid nitrogen, and the starch was purified according to the instructions of Tran et al. (2011). The starch molecules were completely dissolved by the protease, sodium bisulfate, DMSO/LiBr (0.5%) ethanol solution, and the proteins, fats, and non-starch polymers were removed without starch degradation. Starches were further debranched with isoamylase and dissolved in DMSO/LiBr solution and physicochemical properties of starch were identified from the milled rice. The total starches were determined by using the protocols described in our previous study by Yang et al. (2016).

According to the solubility of protein components in different solvents, albumin, globulin, prolamin, and glutelin were extracted with distilled water, dilute hydrochloric acid, ethanol, and dilute alkali in sequence. The biuret colorimetric method was used to determine the remaining species using the Coomassie brilliant blue colorimetric method.

### Determination of Rice Appearance, Milling, Cooking, and Eating Quality

For rice appearance quality, chalk characteristics of brown rice were observed by the cleanliness test-bed according to our previous studies (Tang et al., 2019). Rice grains were milled using a mini universal grinder and dried over a 200-mesh sieve. The length, width, and thickness of brown rice were determined by vernier caliper, and the ratio of length to width were calculate. Three hundred grains of brown rice were randomly selected to detect chalky rice grains and calculate chalky rice rate based on the percentage of chalky rice in the total number. Chalkiness area was determined by the proportion of chalky grain area to total grain area.

Rice milling quality including brown rice rate, milled rice rate, and head rice rate were investigated. During the maturity period, 30 rice panicles with uniform maturity were randomly harvested. After threshing, the grains were naturally dried to the moisture content of 15%. Brown rice percentage (BRP) and milled rice percentage (MRP) were determined by the processing machinery SY88-TH & SY88-TRF (Wuxi Shanglong Grain Equipment Co., Ltd., China), respectively.

Rice cooking and eating quality was determined by the RVA-4500 (a rapid viscometer developed by Newport scientific instruments, Australia). Weigh 3.00 g rice flour with moisture content of about 14.0% into aluminum box, add 25 ml distilled water, and stir it up and down rapidly for 10 times with an agitator to make the rice flour disperse evenly. The determination of RVA characteristic parameters for rice flour was programed as follows: the rice flour solution sample was heated at 50°C for 1 min, then heated to 95°C within 3.8 min, heated at 95°C for 2.5 min, then cooled to 50°C within 3.8 min, and finally heated at 50°C for 1.4 min. The characteristic parameters of RVA spectrum include peak viscosity (PKV), hot paste viscosity (HPV), cooling paste viscosity (CPV), breakdown viscosity (BDV), and depletion value (setback viscosity, SBV) were measured by the viscometer.

### Protein Extraction and Enzymatic Hydrolysis

Lysis buffer (8 M Urea, 40 mM Tris–HCl or tetraethyl-ammonium bromide (TEAB) with 1 mM Phenylmethanesulfonyl fluoride (PMSF), 2 mM Ethylene Diamine Tetraacetic Acid (EDTA) and 10 mM dithiothreitol (DTT), pH 8.5), and two magnetic beads (diameter 5 mm) were used to extract the proteins. The mixtures were placed into a Tissue Lyser for 2 min at 50 Hz to release proteins. After centrifugation at 25,000 g at 4°C for 20 min, the supernatant was transferred into a new tube, reduced with 10 mM DTT at 56°C for 1 h and alkylated by 55 mM iodoacetamide (IAM) in the dark at room temperature for 45 min. Following centrifugation (25,000 g, 4°C, 20 min), the supernatant containing proteins was quantified by Bradford and sodium dodecyl sulfate polyacrylamide gel electrophoresis (SDS-PAGE) 0.100 μg of protein solution per sample and dilute with 50 mM NH_4_HCO_3_ by 4 times volumes. Add 2.5 μg of Trypsin enzyme in the ratio of protein: enzyme = 40:1, and digest for 4 h at 37°C. Add Trypsin once more in the above ratio and continue to digest for 8 h at 37°C. Enzymatic peptides were desalted using a Strata X column and vacuumed to dryness (Gillet et al., 2012; Roest et al., 2014).

### High pH RP Separation

200 ug sample mixtures were diluted with 2 mL of mobile phase A (5% ACN pH 9.8) and injected. The Shimadzu LC-20AB HPLC system coupled with a Gemini high pH C18 column (5 um, 4.6 × 250 mm) was used. Samples were subjected to the column and then eluted at a flow rate of 1 mL/min by gradient: 5% mobile phase B (95% CAN, pH 9.8) for 10 min, 5–35% mobile phase B for 40 min, 35% to 95% mobile phase B for 1 min, flow Phase B lasted 3 min, and 5% mobile phase B equilibrated for 10 min. The elution peak was monitored at a wavelength of 214 nm and component was collected every minute. Components were combined into a total of 10 fractions, which were then freeze-dried.

### DDA Spectral Library and DIA Analysis by Nano-LC-MS/MS

The peptides separated in liquid phase were ionized by a NanoESI source and then placed on the Q-Exactive HF (Thermo Fisher Scientific, San Jose, CA) for DDA and DIA mode detection. The main parameters of DDA were as follows: the ion source voltage was 1.6 kV; the first-order mass spectrum scanning range was 350–1,500 m/z; the resolution was 6,000, the initial m/z of the secondary mass spectrum is fixed at 100; and the resolution is 15,000. The screening conditions for the precursor ions of the secondary fragmentation are the charge 2^+^ to 7^+^, and the peak intensity of more than 10,000 is ranked in the top 20 precursor ions. The fragment ions were detected in an Orbitrap. The dynamic exclusion time was 30 s. The main parameters of DIA were as follows: the ion source voltage was 1.6 kV; the first-order mass spectrum scanning range was 350–1,500 m/z; the resolution was 120,000; and 350–1,500 Da was divided into 40 windows for fragmentation and signal collection. The fragment ions were detected in an Orbitrap. The dynamic exclusion time was 30 s.

### Peptide Detection and Annotation

MaxQuant software was used to complete the identification of DDA for the Mass spectrum RAW data. DIA data was analyzed using Spectronaut, which uses iRT peptides to complete the retention time correction. Then, based on the Target-decoy model applicable to SWATH-MS, the false positive control was completed with FDR 1% to obtain significant quantitative results. data was preprocessed using the MS stats to the, and then the significance test was performed based on the model. Screening was performed according to the Fold change ≥ 2 and *P* < 0.05 as the criteria for determining differentially expressed proteins. The mass spectrometry proteomics data has been deposited to the ProteomeXchange Consortium (http://proteomecentral.proteomexchange.org) via the iProX partner repository with the dataset identifier PXD028032.

### Data Analysis

The identified proteins were classified into three categories (biological process, cellular compartment and molecular function) based on the gene ontology annotation derived from the NCBI database (http://www.ebi.ac.uk/GOA/). Kyoto Encyclopedia of Genes and Genomes (KEGG) database was used to annotate protein pathway (https://www.kegg.jp/). Gene Ontology was used for functional annotation of proteins (http://www.geneontology.org). Data sorting and analysis were performed using Microsoft Excel 2019 and SPSS22 statistical software. Origin 8.1 was employed for figure preparation. Data analysis of variance was analyzed according to a completely random design, and the averages were compared by Duncan's multiple range test (DMRT) based on the least significant difference test at the 5% probability level.

## Conclusions

Our previous studies have shown that elevated temperatures could deteriorate the rice quality through inducing the imbalance ratio of starch and protein components in rice grains. However, due to the complexity of the accumulation process of storage materials, which involves multi-level interactions between gene transcription, translation, protein folding and degradation, thus it is difficult to fully elucidate the mechanism of the effect of elevated temperature on rice quality formation. In this study, we identified a certain number of key proteins (PPROL 14E, PSB28, granule-bound starch synthase I, and 26.7 kda heat shock protein) and metabolic pathways that were sensitive to elevated temperature through DIA quantitative proteomics analysis. The identified differentially expressed proteins were participated in the accumulation of starch and storage protein in the grains, which may be responsible for the deterioration of rice quality under elevated temperature. The results could help to further clarify the potential regulatory mechanism of global warming on rice development and quality formation.

## Data Availability Statement

The original contributions presented in the study are publicly available. This data can be found here: http://proteomecentral.proteomexchange.org/cgi/GetDataset?ID=PXD028032 or https://www.iprox.cn/page/project.html?id=IPX0003400000.

## Author Contributions

ST and YD conceived the experiments. ST designed the experiments. WL performed the experimental work and carried out the proteomics data analysis. XW and KW did the sampling and data analysis. TY and YZ helped in protein isolation. YS provided plant materials. ST and WL wrote the paper. All authors read and approved the final manuscript.

## Funding

This work was supported by the National Key R&D Program, Ministry of Science and Technology, China (Grant No. 2017YFD0300100, 2017YFD0300103 & 2017YFD0300107). This work was also funded by the National Natural Science Foundation of China (Grant No. 32071949 & 31701366). We also received support from the Jiangsu Collaborative Innovation Center for Modern Crop Production (JCIC-MCP) and the Fundamental Research Funds for the Central Universities, China (Grant No. KJQN201802).

## Conflict of Interest

The authors declare that the research was conducted in the absence of any commercial or financial relationships that could be construed as a potential conflict of interest.

## Publisher's Note

All claims expressed in this article are solely those of the authors and do not necessarily represent those of their affiliated organizations, or those of the publisher, the editors and the reviewers. Any product that may be evaluated in this article, or claim that may be made by its manufacturer, is not guaranteed or endorsed by the publisher.
