# Supplementary material for: Effects of High Temperature on Rice Grain Development and Quality Formation Based on Proteomics Comparative Analysis Under Field Warming
Source: Front Plant Sci. 2021 Oct 21;12:746180. doi: 10.3389/fpls.2021.746180 (PMC8566943; doi:10.3389/fpls.2021.746180)

**SUPPLEMENTARY FIGURE LEGENDS**

Supplementary Figure 1: changes of rice canopy temperature and precipitation during the experimental treatment.

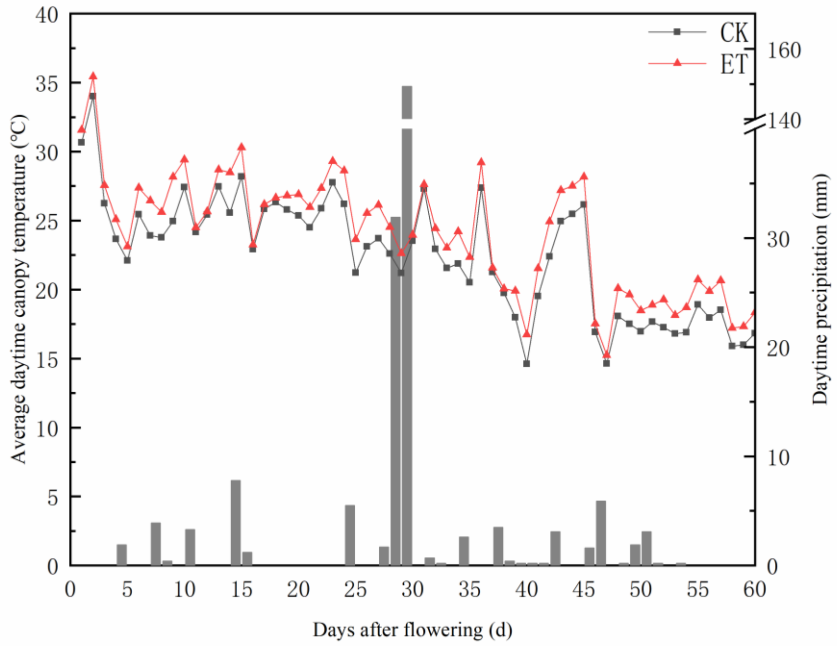


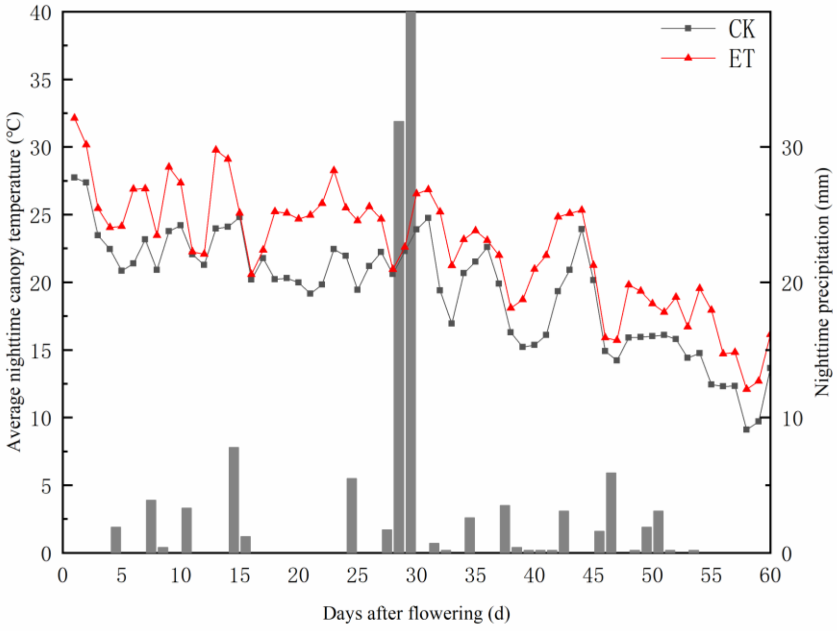

Supplement: Supplementary file 7 [file Image_1.pdf]
